# AS1411 aptamer modified carbon dots via polyethylenimine‐assisted strategy for efficient targeted cancer cell imaging

**DOI:** 10.1111/cpr.12713

**Published:** 2019-11-05

**Authors:** Tingting Kong, Ronghui Zhou, Yujun Zhang, Liying Hao, Xiaoxiao Cai, Bofeng Zhu

**Affiliations:** ^1^ Key Laboratory of Shaanxi Province for Craniofacial Precision Medicine Research College of Stomatology Xi'an Jiaotong University Xi'an China; ^2^ Clinical Research Center of Shaanxi Province for Dental and Maxillofacial Diseases College of Stomatology Xi'an Jiaotong University Xi'an China; ^3^ State Key Laboratory of Oral Diseases West China Hospital of Stomatology Sichuan University Chengdu China; ^4^ Department of Prosthodontics School and Hospital of Stomatology Shandong University & Shandong Provincial Key Laboratory of Oral Tissue Regeneration & Shandong Engineering Laboratory for Dental Materials and Oral Tissue Regeneration Jinan China; ^5^ Department of Forensic Genetics School of Forensic Medicine Southern Medical University Guangzhou China

**Keywords:** AS1411 aptamer, carbon dots, polyethylenimine, targeted imaging

## Abstract

**Objectives:**

Carbon dots (CDs), as a fascinating class of fluorescent carbon nanomaterials, have been proven to be powerful tools in the field of bioimaging and biosensing due to their small size, suitable photostability and favourable biocompatibility. However, the cellular uptake of free CDs lacks selectivity and the same negative charges as cell membranes may cause inefficient cell internalization. In this study, an efficient detecting and targeting nanosystem was developed based on the DNA aptamer AS1411 modified CDs with polyethyleneimine (PEI) as connecting bridge.

**Materials and methods:**

Hydrothermally prepared CDs were assembled with positive‐charged PEI, followed by conjugation with AS1411 through electrostatic interaction to form CDs‐PEI‐AS1411 nanocomplexes. The CDs, CDs‐PEI and CDs‐PEI‐AS1411 were characterized by transmission electron microscopy (TEM), fourier transform infrared (FTIR) spectra, UV–vis spectra, zeta potential measurements and capillary electrophoresis characterizations. The cytotoxicity investigation of the CDs‐PEI‐AS1411 and CDs‐PEI in both MCF‐7 and L929 cells was carried out by the CCK‐8 assay. The cellular uptake of the CDs‐PEI‐AS1411 was studied with confocal microscopy and flow cytometry.

**Results:**

The as‐prepared nanosystem possessed good photostability and no obvious cytotoxicity. On the basis of the confocal laser scanning microscope observation and the flow cytometry studies, the cellular uptake of CDs‐PEI‐AS1411 nanosystem in MCF‐7 cells was significantly higher than that of L929 cells, which revealed the highly selective detection ability of nucleolin‐positive cells.

**Conclusions:**

The results of this study indicated that the CDs‐PEI‐AS1411 nanosystem had a potential value in cancer cell targeted imaging.

## 1 INTRODUCTION

Carbon dots (CDs) have received great attention in bio‐related applications such as biosensing and bioimaging due to their small size, low toxicity, good biocompatibility and good stability. Meanwhile, CDs modified with small oligonucleotide ligands (aptamer), peptides or small molecules have been widely used in targeted fluorescent imaging, drug delivery and cancer therapy.^1^, ^2^, ^3^, ^4^, ^5^, ^6^ Among the numerous targeted agent, aptamer possessed chemical stability, non‐immunogenicity and easy synthesis, which garnered a lot of attention.^7^, ^8^, ^9^, ^10^ It is well known that AS1411 is a common aptamer with 26‐mer oligonucleotide, which can bind to the nucleolin that is highly expressed on the surface of most cancer cells.^11^ As a result, AS1411 is a promising candidate for precisely targeting.^12^, ^13^, ^14^, ^15^, ^16^ However, for AS1411 favoured fluorescence targeting imaging, the relatively complex strategy of chemical coupling is usually adopted to connect imaging agent with AS1411, which may result in poor targeting ability. Besides, the imaging agent conjugated with negative charged aptamer AS1411 always exhibit the same negative charge as cell membrane, which may affect the efficiency of cellular uptake ascribe to the electrostatic repulsion.

Polymer‐assisted surface functionalization can easily provide a high surface with abundant functional groups and facilitate attachment of other substances.^17^ Polyethylenimine (PEI) is a cationic polymer material containing a large number of amino groups, and widely used in the field of biological transfer due to its high transfer efficiency and low cost.^18^, ^19^ In addition, the use of PEI as scaffolds for loading of nanomaterials has been shown as an effective way for facilitating cellular uptake efficiency of these materials, such as quantum dots, Fe_3_O_4_ magnetic nanoparticles.^20^, ^21^, ^22^ Furthermore, surface functionalization with PEI can give CDs strong positive surface charges, which is beneficial to encapsulating some negatively charged macromolecules, such as DNA.^23^, ^24^ Tian et al^25^ combined PEI and tetrahedral DNA nanostructures (TDNs) through electrostatic interaction to form novel drug‐delivery vehicles with enhanced systemic stability, cell‐entry ability and lysosome‐escape ability. Furthermore, the high density of amino groups in PEI not only generate plenty of attachment sites, but also render the CDs with good colloidal stability.^26^, ^27^

**Scheme 1:**
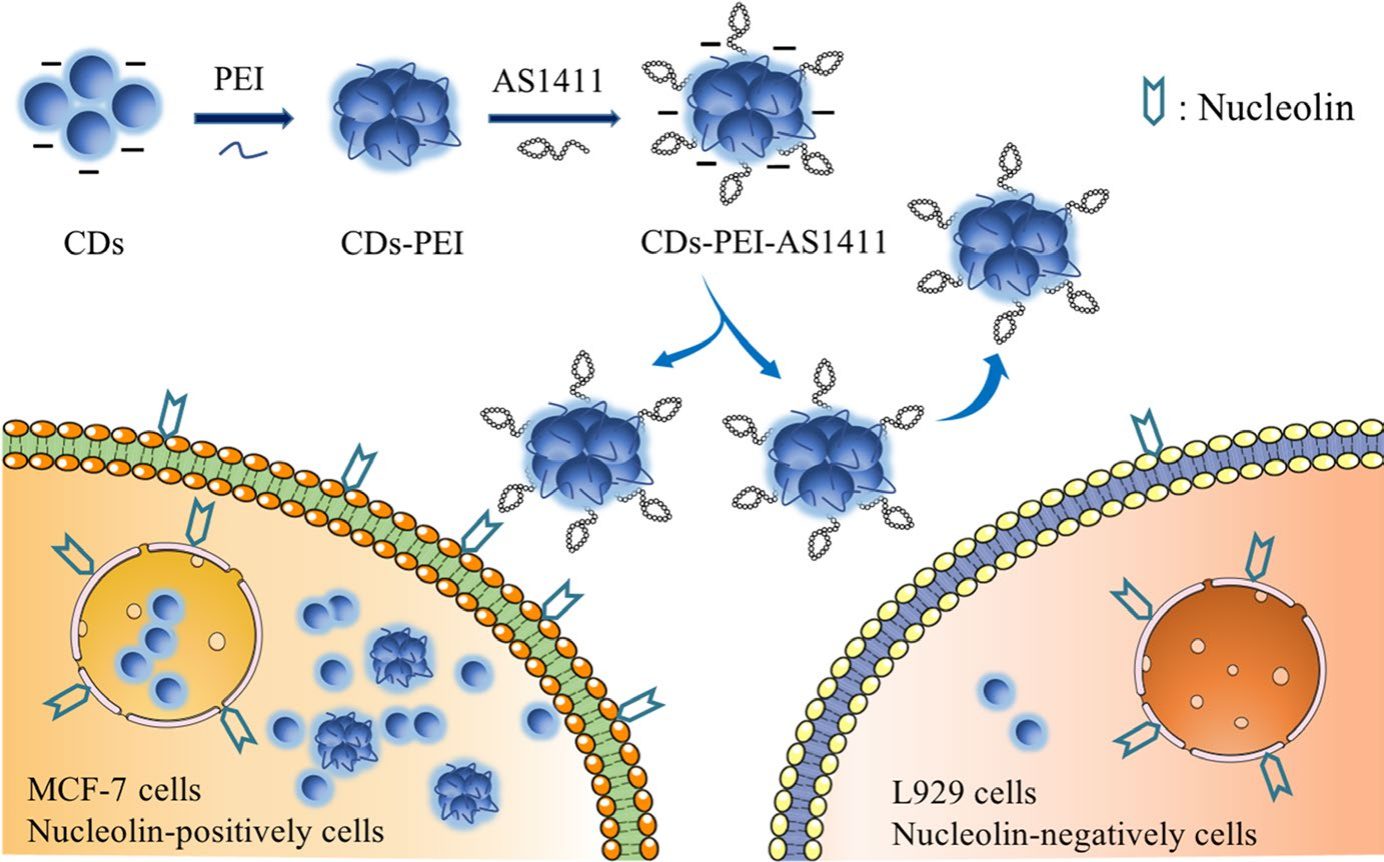
Predicted mechanism of the different cellular uptake behaviour of CDs‐PEI‐AS1411 with the nucleolin‐positive MCF‐7 cancer cells and nucleolin‐negative L929 fibroblast cells

Herein, a fluorescence imaging nanosystem based on AS1411 modified PEI twisted CDs was developed for targeted imaging of nucleolin highly expressed cancer cells (Scheme 1). High brightness CDs were first synthesized from citric acid through hydrothermal process, and then coated with PEI to form the CDs‐PEI assembly. Finally, the CDs‐PEI assembly was further attached with aptamer AS1411 via electrostatic interaction. To investigate the targeting ability of the developed CDs‐PEI‐AS1411 nanosystem to cancer cells, the interaction between CDs‐PEI‐AS1411 and MCF‐7 (overexpressed nucleolin) or L929 (low nucleolin expression) cells were studied through cell imaging and flow cytometry. The results showed that CDs‐PEI‐AS1411 nanosystem possessed higher cellular uptake efficiency in MCF‐7 cells than L929 cells. For cancer MCF‐7 cells, the high affinity between AS1411 and the relevant overexpressed receptor nucleolin led the aptamer release, and the surface charge of nanosystem rise at the same time, resulting in enhanced cellular uptake. By employing the AS1411 aptamer, enhanced selectivity and sensitivity were achieved.

## 2 MATERIALS AND METHODS

### 2.1 Materials

Ethylenediamine and citric acid were purchased from Aladdin Chemical Inc, and polyethylenimine (PEI, MW = 25 000) was from Sigma‐Aldrich Chemical Co., Ltd. Centrifugal filter (MWCO = 30 kDa) was obtained from Millipore Ltd. AS1411 aptamer (5′‐GGTGGTGGTGGTTGTGGTGGTGGTGG‐3′) was synthesized by Tsingke Biotech Co., Ltd. Human breast adenocarcinoma cell line (MCF‐7) and mouse fibroblast cell line (L929) were provided from the Key Laboratory of Oral Diseases of Sichuan University (Chengdu, China). Foetal bovine serum (FBS), penicillin‐streptomycin, Dulbecco's modified Eagle medium (DMED), RPMI 1640 medium, 0.25% trypsin‐EDTA and phosphate buffer saline (PBS) were obtained from GIBCO, Invitrogen Co. The cells were cultured in a humidified incubator at 37°C in the atmosphere of 5% CO_2_. Cell counting Kit‐8 (CCK‐8) and FITC‐labelled phalloidin were supplied from Shanghai Dojindo Technology Chemical Corp. and Sigma‐Aldrich Chemical Co., Ltd, respectively.

### 2.2 Preparation of CDs

In a typical synthesis, citric acid (1.05 g) and ethylenediamine (335 µL) were dissolved in 10 mL deionized water. Then the mixed solution was transferred to an autoclave and heated at 200°C for 5 hours. After the product cooled down to room temperature, the product was purified with column chromatography (with water as eluent) to obtain the uniform‐sized particles.^28^, ^29^, ^30^ Finally, the obtained CDs were light yellow and transparent, which were stored at 4°C for further treatment.

### 2.3 Preparation of CDs‐PEI and CDs‐PEI‐AS1411

In order to obtain CDs‐PEI, 15 µL PEI (2 mg/mL) was mixed with 100 µL CDs solution (4 mg/mL) and 85 µL deionized water, and then stirred overnight at room temperature. For CDs‐PEI‐AS1411, different volumes of AS1411 (10‐50 µL, 100 µmol/L) were mixed with the above CDs‐PEI solution with gently stirring at 4°C for 2 hours. After that, free DNA was removed using centrifugal filtration (MWCO = 30 kDa) at 4000 rpm for 10 minutes, and then the fluorescence intensities were recorded to determine the proper CDs/aptamer ratios. CDs‐PEI‐AS1411 were finally collected and kept at 4°C for later experiments.

### 2.4 Characterizations of CDs, CDs‐PEI and CDs‐PEI‐AS1411

Surface morphology analyses of particles were performed using a Tecnai G2 F20 transmission electron microscope (TEM, FEI). Particle size and zeta potential measurements were carried out with a Nano‐ZS90 Zetasizer. The fourier transform infrared (FTIR) spectra were characterized using a 5DX FT‐IR spectrometer (Nicolet). The UV‐vis spectra were measured by a U‐3900H UV‐vis spectrophotometer (Hitachi), and the fluorescence spectra were recorded by a RF‐5301PC photoluminescence spectrometer (Shimadzu). Capillary electrophoresis analyses were performed on a Qsep100 Bio‐Fragment Analyzer (BiOptic). Fluorescence lifetime was recorded using a PPD‐850 fluorescence‐photon counting detector (HORIBA, Japan).

### 2.5 Confocal laser scanning microscope observation

Confocal laser scanning microscopy was employed to observe the cellular uptake of the CDs‐PEI‐AS1411. MCF‐7 and L929 cells were seeded in confocal microscope dishes at 2 × 10^4^ cells per well and incubated overnight. Then, the CDs‐PEI‐AS1411 were added to the above cells at the CDs concentration of 200 µg/mL. After different treatment time (6, 12, 24 and 48 hours), the supernatant was removed and the cells were fixed with 4% paraformaldehyde. Subsequently, the cell cytoskeleton was stained with FITC‐labelled phalloidin and the cells were imaged by a Nikon AIR‐MP confocal laser scanning microscope (Japan).

### 2.6 Flow cytometry analysis

The cellular uptake of the CDs‐PEI‐AS1411 and the CDs‐PEI was further studied with flow cytometry. MCF‐7 and L929 cells were cultured overnight at 2 × 10^5^ cells per well in 6‐well plates. After that, the cells were treated with CDs‐PEI‐AS1411 or CDs‐PEI (the CDs concentration of 200 µg/mL) for 6 or 12 hours. Then, the cells were washed for three times with PBS and centrifuged at 1000 rpm for 5 minutes. After resuspended in 500 µL PBS, the cells were detected by a Coulter FC500 flow cytometer (Beckman).

### 2.7 Cytotoxicity assay

The cytotoxicity of the CDs‐PEI‐AS1411 and CDs‐PEI was investigated by the CCK‐8 assay. MCF‐7 cells and L929 cells were seeded (5000 cells per well) in 96‐well plates and grew overnight. And then, the cells were replaced with fresh medium containing different concentrations of CDs‐PEI‐AS1411 or equivalent concentrations of CDs‐PEI. After incubation for 24 hours, the CCK‐8 reagent (10%) was added to each well to test the cell viability through absorbance measurement at a wavelength of 450 nm with a VariOskanFlas 3001 microplate reader (Thermo).

### 2.8 Statistical analysis

Statistical significance of the data was analysed through independent sample *t* test by SPSS Statistics R24.0 software (IBM). Data are presented as mean result ± SD. Probability value of <.05 was considered statistically significant (**#** *P* < .05, *P < .05, **P < .01).

## 3 RESULTS

### 3.1 Characterization

**Figure 1:**
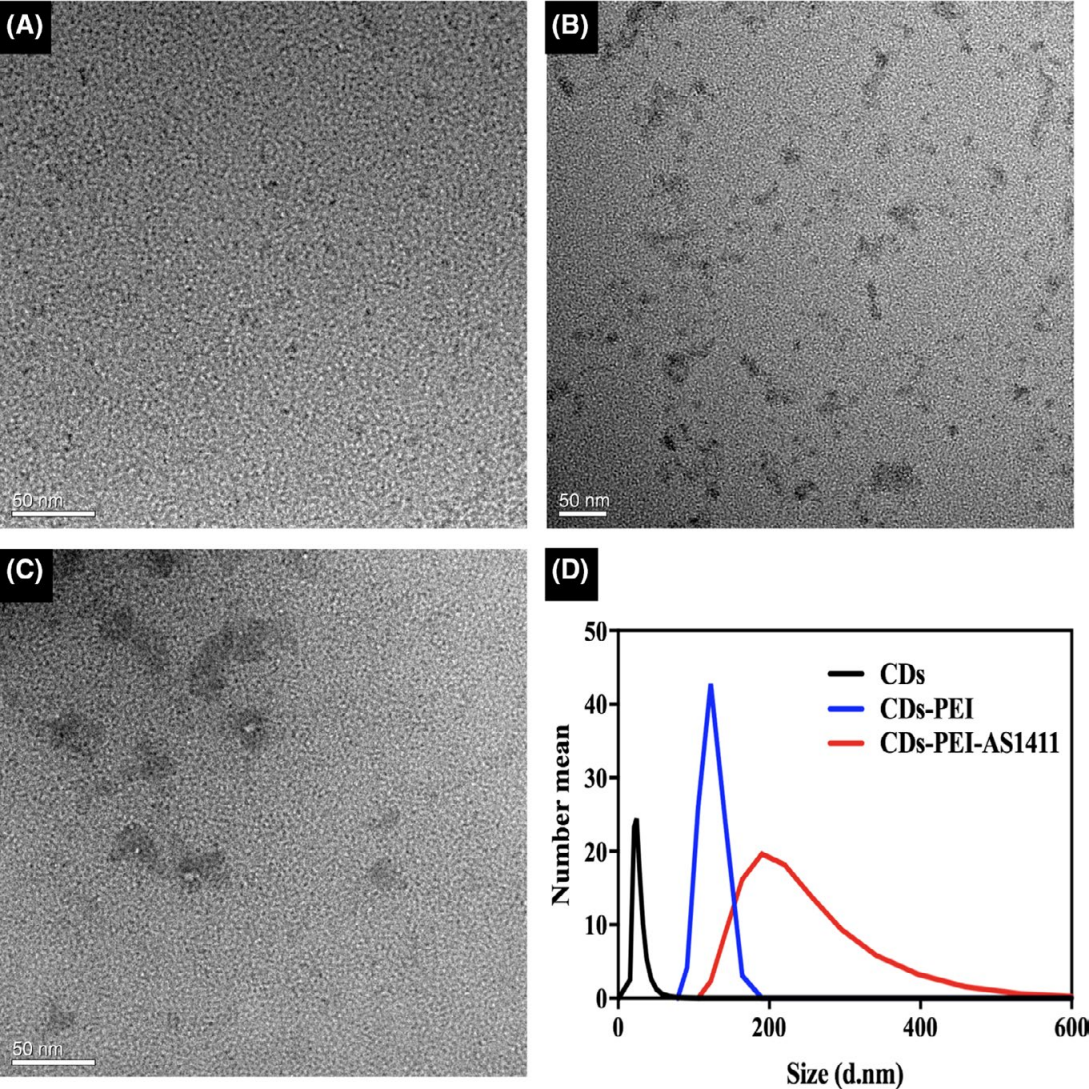
Transmission electron microscopy images of CDs (A), CDs‐PEI (B) and CDs‐PEI‐AS1411 (C). (D) Particle size distribution of CDs, CDs‐PEI and CDs‐PEI‐AS1411. Scale bar = 50 nm

Surface morphology of the CDs, CDs‐PEI and CDs‐PEI‐AS1411 were characterized by TEM. As shown in Figure 1A, the CDs were well‐dispersed and quasi‐spherical in shape. While, after treated with PEI, the formed CDs‐PEI were aggregated into linear chain (Figure 1B), which was consistent with the characterization of previous study.^19^ Subsequently, with further assembly of AS1411 onto the CDs‐PEI, the complexes tended to form larger clusters (Figure 1C), indicating the successful preparation of CDs‐PEI‐AS1411 via electrostatic interaction. Moreover, the assembly of CDs‐PEI‐AS1411 was also demonstrated by dynamic light scattering measurement (Figure 1D). The particle sizes of free CDs and CDs‐PEI were 26.19 nm and 122.7 nm, respectively. Non‐covalent conjugation of AS1411 aptamer to CDs‐PEI enlarged its size to 227.1 nm, which was consistent with the above TEM images.

**Figure 2:**
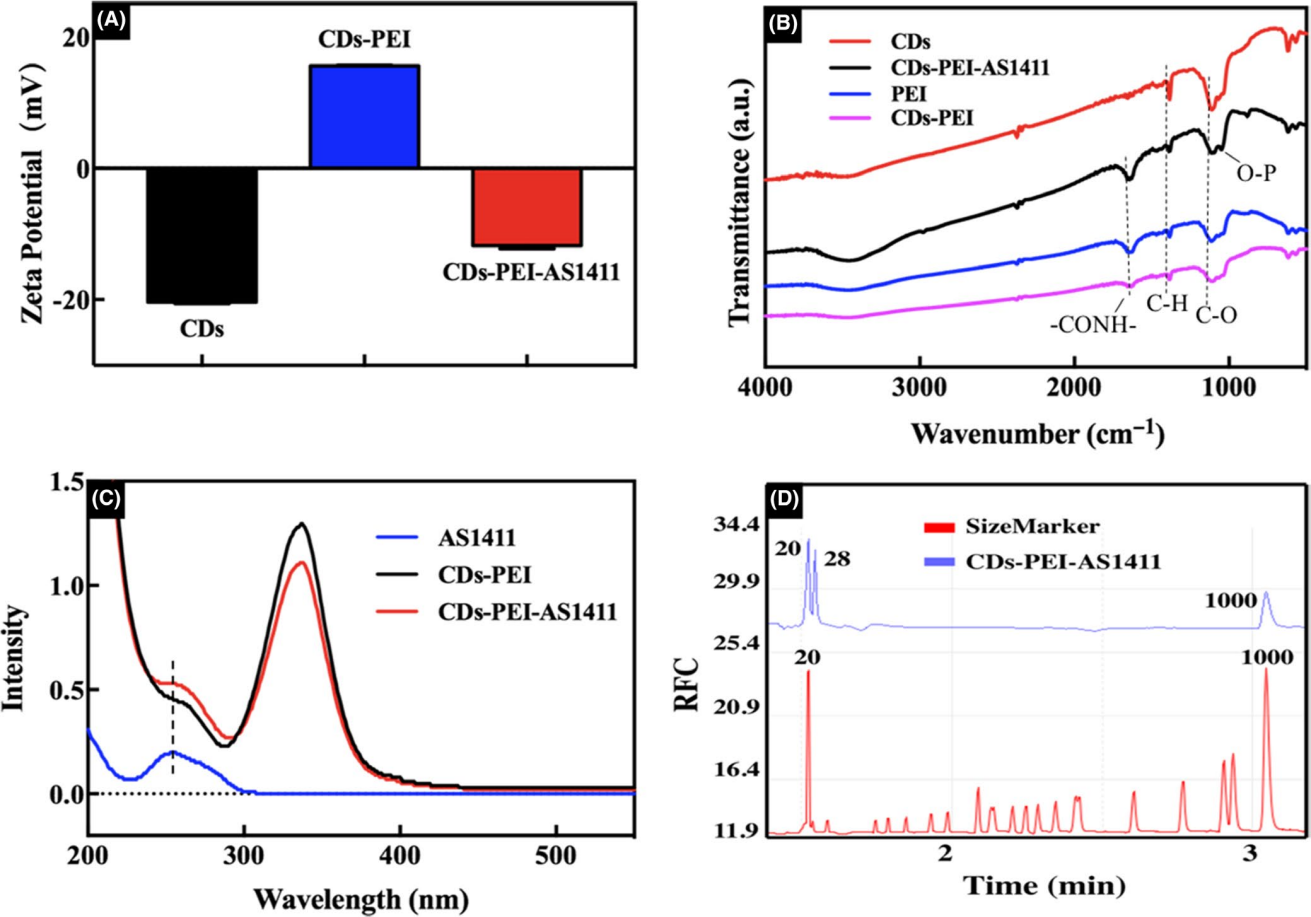
(A) Zeta potential values of CDs, CDs‐PEI and CDs‐PEI‐AS1411. (B) FTIR spectra of CDs, PEI, CDs‐PEI and CDs‐PEI‐AS1411. (C) UV‐vis spectra of AS1411, CDs‐PEI and CDs‐PEI‐AS1411. (D) Electropherograms of size marker and CDs‐PEI‐AS1411

To further confirm the successful preparation of CDs‐PEI‐AS1411 nanosystem, zeta potentials of the CDs, CDs‐PEI and CDs‐PEI‐AS1411 were first investigated, which was displayed in Figure 2A. The average zeta potential value of the CDs was −20.3 mV and raised to +15.6 mV after PEI modification, which was attributed to the addition of the positively charged amino groups. Consequently, the positive charge facilitated the assembly of the CDs‐PEI with negatively charged aptamer of AS1411 through electrostatic interaction. Additionally, the charge of the CDs‐PEI‐AS1411 reversed back to −11.8 mV, which further confirmed the successful fabrication of the complexes. Meanwhile, the FTIR spectra provided further understanding on the structure of the formed nanoparticles. As presented in Figure 2B, the peaks at 1384 cm^−1^ and 1109 cm^−1^ were attributed to C‐H and C‐O, respectively. Compared with the CDs, for CDs‐PEI, a typical absorption peak belonging to the – CONH – emerged at 1700 cm^−1^,^31^ which indicated the successful coating of PEI via amide linkages. The newly formed surface functional groups improved the stability and hydrophilicity of the CDs. In addition, after DNA capping, the CDs‐PEI‐AS1411 complexes showed characteristic absorption bands at 1050 cm^−1^ which could be assigned to the O‐P stretching vibration of phosphate groups.^32^ These characteristic results confirmed the achievement of the CDs‐PEI‐AS1411. Significantly, the combination between AS1411 and the CDs‐PEI was further verified by the measurement of UV/Vis absorption spectra and capillary electrophoresis. As illustrated in Figure 2C, 2 typical UV‐vis absorbance peak of the AS1411 at 260 nm could be observed in the CDs‐PEI‐AS1411.^33^ Furthermore, in the capillary electrophoresis (Figure 2D), a peak at the molecular weight of 28 bp was detected when AS1411 was non‐covalently attached to the CDs‐PEI, which indicated the successful capping of DNA molecules on the nanoparticles.

**Figure 3:**
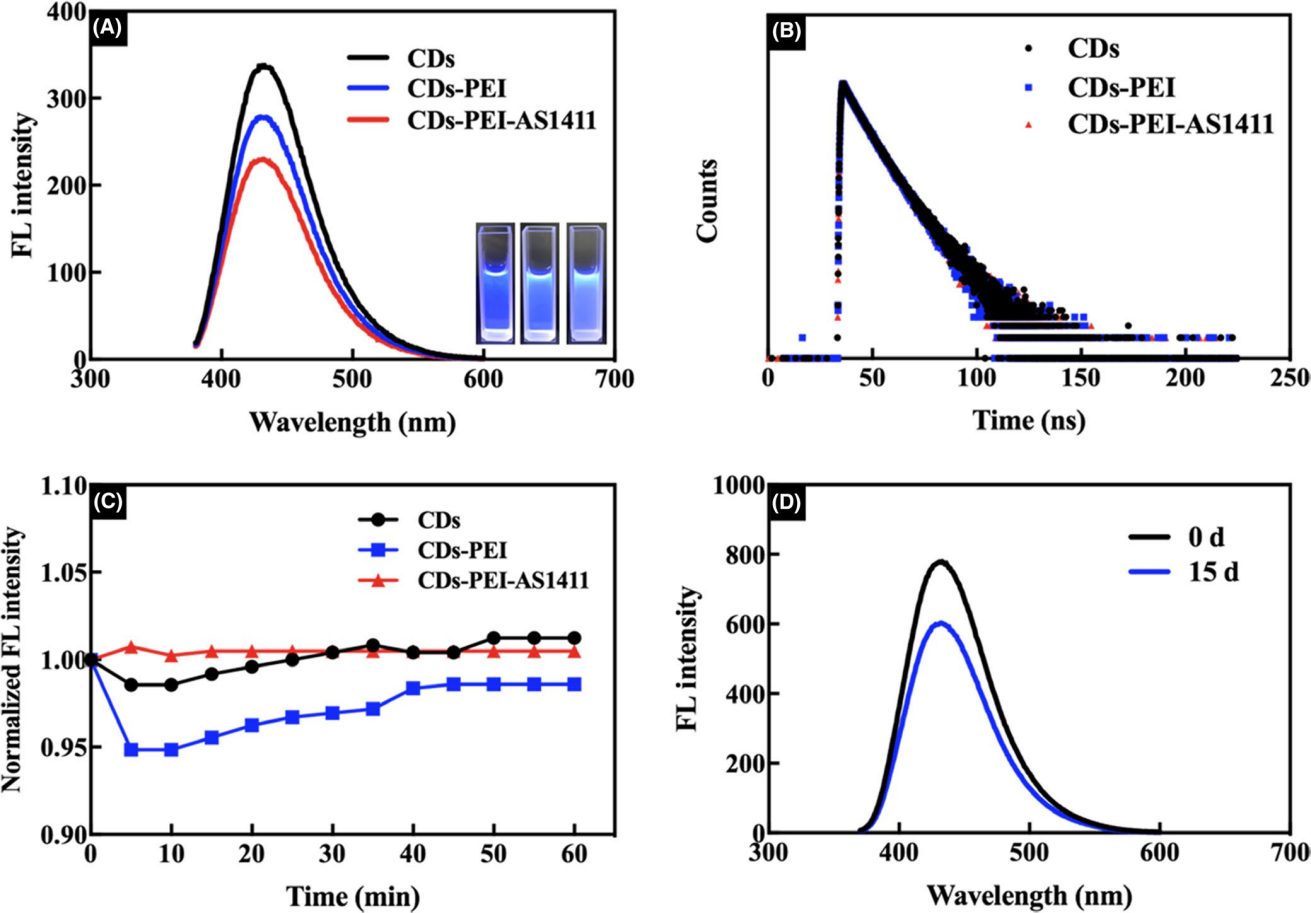
(A) Fluorescence spectra of CDs, CDs‐PEI and CDs‐PEI‐AS1411. Inset: optical images of CDs (left), CDs‐PEI (middle) and CDs‐PEI‐AS1411 (right) in aqueous solution under UV light. (B) The fluorescence decay curves of CDs, CDs‐PEI and CDs‐PEI‐AS1411. (C) Fluorescent intensity of CDs‐PEIAS1411 under 360 nm irradiation at different intervals of time. (D) Fluorescence profiles of CDs‐PEI‐AS1411 in water showing aqueous stability up to 15 d preservation time

To explore the optical properties, the photoluminescence (PL) emission spectra of the CDs, CDs‐PEI and CDs‐PEI‐AS1411 nanoparticles were first investigated, which was shown in Figure 3A. The CDs exhibited strong emission peak under excitation wavelength of 360 nm. With the assembly of the PEI and the DNA aptamer, both the CDs‐PEI and CDs‐PEI‐AS1411 showed a slight decrease, but would not affect their imaging application. Moreover, the inset image of the CDs(left), CDs‐PEI (middle) and CDs‐PEI‐AS1411 (right) showed similar bright blue luminescence under the UV light. Previous study showed that aptamer molecules can partially inhibit the fluorescence of CDs.^4^ Therefore, in order to maintain their PL property, the fluorescence intensities of CDs‐PEI‐AS1411 were tested with different CDs/aptamer weight ratios. As shown in Figure S1, the complexes at CDs/aptamer ratio of 16.7:1 exhibited the highest fluorescence intensity, and we thus chose this ratio for subsequent experiments. Then the fluorescence decay curves (Figure 3B) of the CDs, CDs‐PEI and CDs‐PEI‐AS1411 were also measured and no significant difference in fluorescence lifetime was found. The high photostability is the key factor of the fluorescent nanomaterials for their biological applications, especially for biological imaging. Herein, we studied the photostability of the CDs, CDs‐PEI and CDs‐PEI‐AS1411, which was exposed under excitation of 360 nm for various amounts of time (Figure 3C). For the CDs and CDs‐PEI, the fluorescence intensity decreased only slightly at the beginning (5 min) and then showed a rising tendency in the following irradiation process. As to the CDs‐PEI‐AS1411, the fluorescence intensity preserved almost 100% of the initial intensity after 60 min irradiation, indicating the high photostability of the resultant complexes. Meanwhile, the aqueous stability of the CDs‐PEI‐AS1411 was also investigated. From the fluorescence profile (Figure 3D), it was observed that the CDs‐PEI‐AS1411 still exhibited good aqueous stability after storage even for 15 days. The emission wavelength of the CDs‐PEI‐AS1411 did not shift and the fluorescent intensity changed little. Consequently, our as‐prepared nanoparticles with good photostability and aqueous stability were the suitable candidates for the biological imaging applications.

### 3.2 Cytotoxicity assay

**Figure 4:**
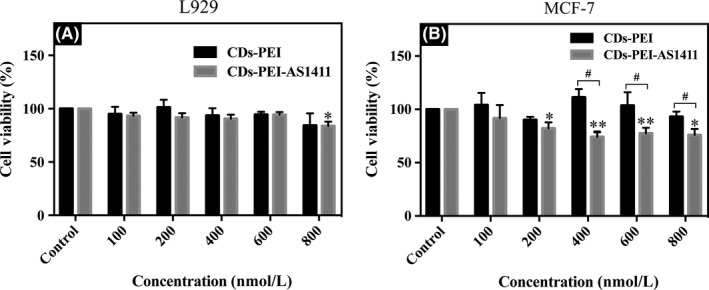
Cell viability of L929 cells (A) and MCF‐7 cells (B) exposed to various concentrations of CDs‐PEI‐AS1411 (at the AS1411 concentrations of 100, 200, 400, 600 and 800 nmol/L) and the relative amounts of CDs‐PEI for 24 h treatment. *P < .05, **P < .01 vs control; #*P *< .05 vs CDs‐PEI‐treated group. Data are shown as mean ± SD. (n = 3)

In order to evaluate the cytotoxicity of the obtained nanoparticles CDs‐PEI and CDs‐PEI‐AS1411, the cell viability studies based on CCK‐8 assay were performed in normal L929 cells (Figure 4A) and cancer MCF‐7 cells (Figure 4B) for 24 hours. The CDs‐PEI displayed limited effect on cell viability in both cell lines with a viability of above 84% at all studied doses. After the treatment with the CDs‐PEI‐AS1411, the cell viability of the L929 cells was similar to that of CDs‐PEI. While in MCF‐7 cells, the CDs‐PEI‐AS1411 showed enhanced cytotoxicity, and significant differences in the viability between the CDs‐PEI and the CDs‐PEI‐AS1411 emerged with the increased concentration from 400 to 800 nmol/L.

### 3.3 Intracellular uptake

**Figure 5:**
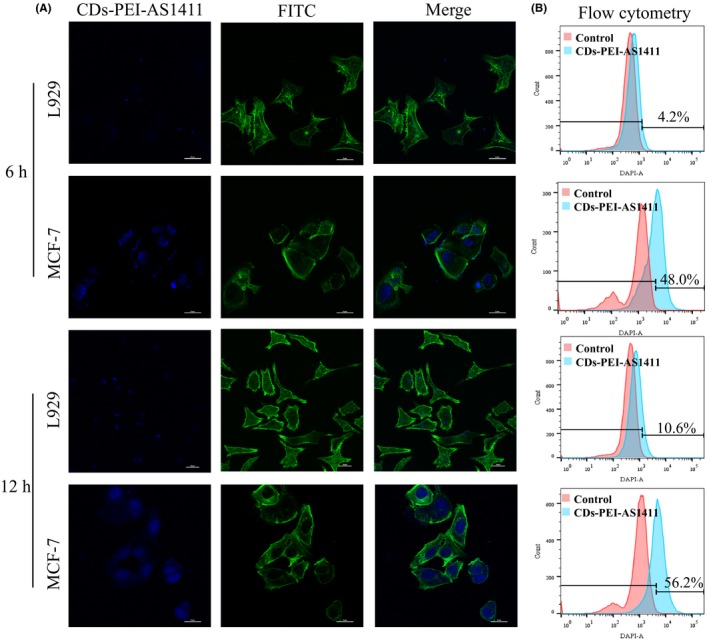
Intracellular uptake analyses. (A) Confocal microscopy images of MCF‐7 cells and L929 cells incubated with the CDs‐PEI‐AS1411 for 6 h and 12 h, respectively (blue, CDs; green, cytoskeleton stained with FITC‐labelled phalloidin). (B) Flow cytometry profiles of MCF‐7 cells and L929 cells treated with the CDs‐PEI‐AS1411 for 6 h and 12 h, respectively

It is known that AS1411 can bind to nucleolin specifically and then subsequently penetrate into the tumour cell. Nucleolin is known to be highly expressed in MCF‐7 cells, but rarely expressed in L929 cells.^13^ In this study, we investigated the potential of the CDs‐PEI‐AS1411 complexes for bioimaging and targeting cancer cells with the MCF‐7 cells and L929 cells. First, confocal microscopy analysis was employed to evaluate the cell internalization efficiency of the CDs‐PEI‐AS1411 (Figure 5A). After 6 hours of incubation, the MCF‐7 cells exhibited clear internalization of the CDs‐PEI‐AS1411 giving bright blue fluorescence, while the L929 cells emitted particularly weak fluorescence. After treatment for 12 hours, the blue fluorescence became extremely strong in the MCF‐7 cells and showed a trend of nuclear accumulation. For the L929 cells, limited fluorescence increase was observed, which indicated the weak penetration of the CDs‐PEI‐AS1411. Moreover, after further incubation of the CDs‐PEI‐AS1411 for 24 hours and 48 hours (Figure S2), the MCF‐7 cells still showed strong blue fluorescence, especially in cell nucleus, which indicated good intracellular stability and long‐lasting fluorescence imaging property of the CDs‐PEI‐AS1411. In contrast with the MCF‐7 cells, the L929 cells showed an almost negligible fluorescence.

Next, flow cytometry results demonstrated the cell uptake of the CDs‐PEI and CDs‐PEI‐AS1411 in the MCF‐7 cells and the L929 cells. As depicted in Figure 5B, the cell uptake ratios of CDs‐PEI‐AS1411 in the MCF‐7 cells were 48.0% and 56.2% after 6 hours and 12 hours of treatment, respectively, which was approximately 11‐fold and 5.5‐fold higher than that of the L929 cells. For CDs‐PEI, the uptake ratio in L929 cells and MCF‐7 cells were similar, 42.6% and 46.4%, respectively, after 6 hours of incubation, and 50.5% and 48.5%, respectively, after 12 hours of incubation (Figure S3).

## 4 DISCUSSION

In present study, we synthesized a targeting nanoprobe for nucleolin highly expressed cancer cells labelling based on CDs which have been successfully prepared in previous experiments with favourable biocompatibility and fluorescence properties.^34^ As the bridge between CDs and the aptamer AS1411, PEI showed good biocompatibility in both L929 and MCF‐7 cells, while, the nanosystem exhibited enhanced cytotoxicity in MCF‐7 cells after loading AS1411. The negatively charged AS1411 might shield the cytotoxicity through preventing strong interactions with the normal cells,^35^ and therefore the L929 cell still maintained high cell viability after culture with the CDs‐PEI‐AS1411. With the enhanced cell internalization of the CDs‐PEI‐AS1411 nanosystem in the MCF‐7 cells, the shielding effect by the negatively charged AS1411 was reduced due to the high affinity between AS1411 and nucleolin receptors. It might be inferred that the significant differences in cytotoxicity were related to the different cell internalization of the nanoparticles.

Then, we studied the cell uptake of the CDs‐PEI‐AS1411 nanosystem in both cell lines. From the confocal microscopy imaging, the fluorescence of MCF‐7 cells was significantly brighter than that of L929 cells after being treated with the CDs‐PEI‐AS1411. The different results might be due to different levels of nucleolin expression in the two cells. The cell uptake process was more efficient in the cells with higher nucleolin expression. The specific interactions between the AS1411 of the CDs‐PEI‐AS1411 and nucleolin receptors in the MCF‐7 cell membrane probably favoured the highly efficient cell internalization. More importantly, due to the high affinity between AS1411 and the nucleolin receptors, the aptamer released from the CDs‐PEI and subsequently the surface charge changed from negative to positive, which significantly facilitated cell uptake process. Besides, the flow cytometry results were consistent with the above cell imaging analyses, which implied our CDs‐PEI‐AS1411 possessed high cell uptake efficiency and good cancer cell selectivity capacity.

## 5 CONCLUSIONS

In summary, we developed a surface charge inversion nanosystem for specific targeted imaging using CD nanoparticle probes labelled with DNA aptamer AS1411. In our system, the hydrothermally synthesized CDs were first wrapped with PEI and then modified with AS1411 through electrostatic interaction. The negatively charged CDs‐PEI‐AS1411 nanocomplexes showed less cellular uptake and good biocompatibility for L929 cells. While for MCF‐7 cells, the presence of nucleolin on the membrane made the AS1411 release from CDs‐PEI and consequently led the surface charge reverse. The exposed CDs‐PEI with rising surface charge exhibited efficient cellular uptake. Due to AS1411 aptamer specific identification and the surface charge inversion, the CDs‐PEI‐AS1411 nanosystem targeted the MCF‐7 cells with high affinity and specificity. We believe our as‐proposed nanosystem has a powerful potential for highly selective and sensitive detection of nucleolin‐positive cells.

## CONFLICTS OF INTEREST

There are no conflicts of interests to declare.

## AUTHOR CONTRIBUTION

Ronghui Zhou, Xiaoxiao Cai and Bofeng Zhu designed the experiments. Tingting Kong and Liying Hao performed the experiments. Yujun Zhang analysed the data. Tingting Kong wrote the manuscript. Xiaoxiao Cai and Bofeng Zhu critically revised the manuscript.

## Data Availability

The data that support the findings of this study are available from the corresponding author upon reasonable request.
