# Supplementary material for: AS1411 aptamer modified carbon dots via polyethylenimine‐assisted strategy for efficient targeted cancer cell imaging
Source: Cell Prolif. 2019 Nov 5;53(1):e12713. doi: 10.1111/cpr.12713 (PMC6985679; doi:10.1111/cpr.12713)
Supplement: Supplementary file 1 [file CPR-53-e12713-s001.docx]

**Supporting information**

**AS1411 aptamer modified carbon dots via polyethylenimine-assisted strategy for efficient targeted cancer cell imaging**

Tingting Kong^1,2,3#^, Ronghui Zhou^3^^#^, Liying Hao^3^, Xiaoxiao Cai^3*^, Bofeng Zhu^1,2,4^^*^

^1^Key Laboratory of Shaanxi Province for Craniofacial Precision Medicine Research, College of Stomatology, Xi’an Jiaotong University, Xi’an, P. R. China

^2^Clinical Research Center of Shaanxi Province for Dental and Maxillofacial Diseases, College of Stomatology, Xi’an Jiaotong University, Xi’an, P. R. China

^3^State Key Laboratory of Oral Diseases, West China Hospital of Stomatology, Sichuan University, Chengdu, P. R. China

^4^Department of Forensic Genetics, School of Forensic Medicine, Southern Medical University, Guangzhou, P. R. China

^#^The first two authors were equal to this article

^*^These authors contributed equally to this work and were co-corresponding authors

***Correspondence:**

Bofeng Zhu, Key Laboratory of Shaanxi Province for Craniofacial Precision Medicine Research, College of Stomatology, Xi’an Jiaotong University, Xi’an 710004, PR China. E-mail: [zhubofeng7372@126.com](mailto:zhubofeng7372@126.com)


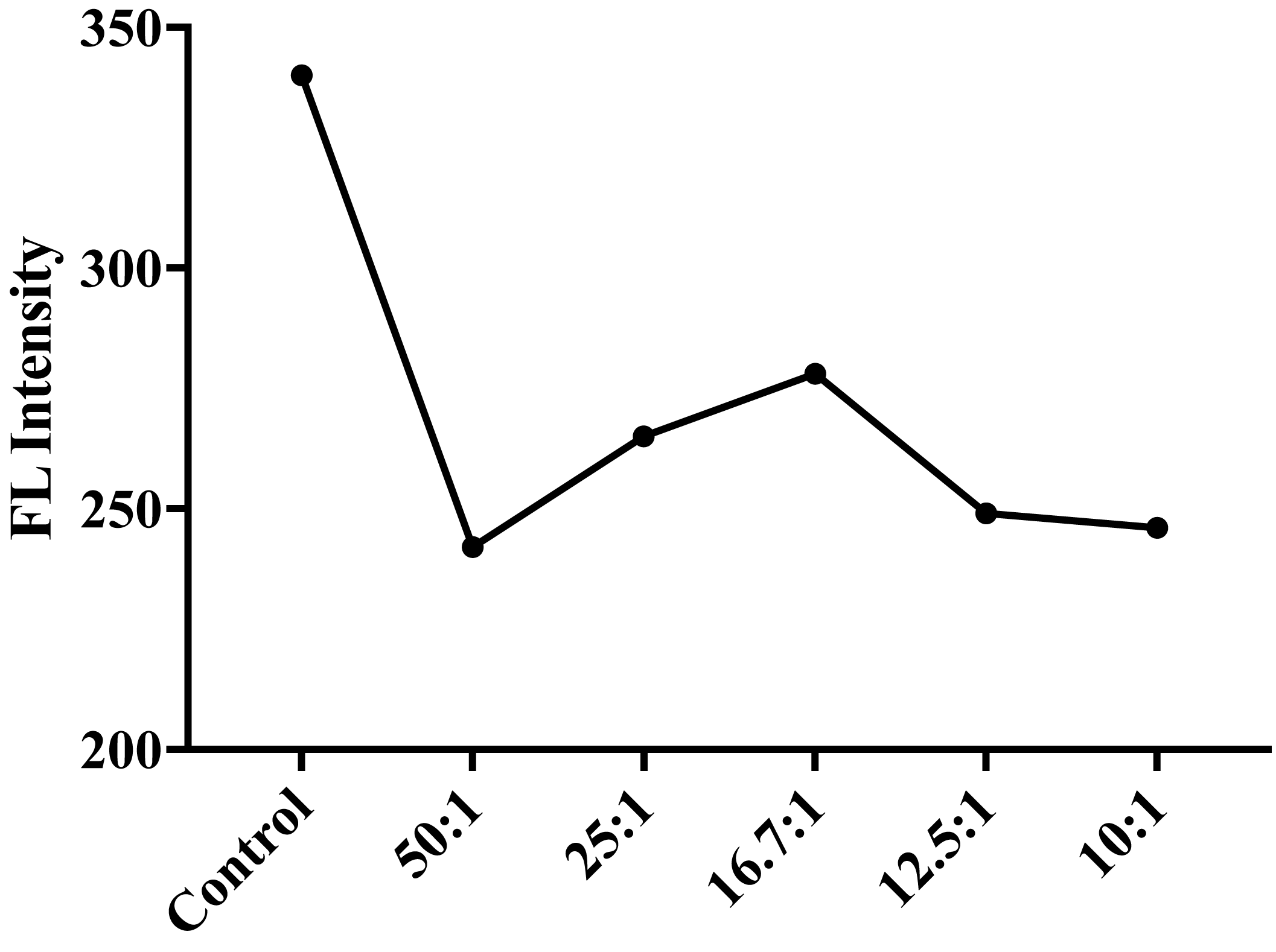


**Figure S1.** Fluorescent intensity of CDs-PEI-AS1411 with different CDs/aptamer ratios (λ_ex_=360 nm).


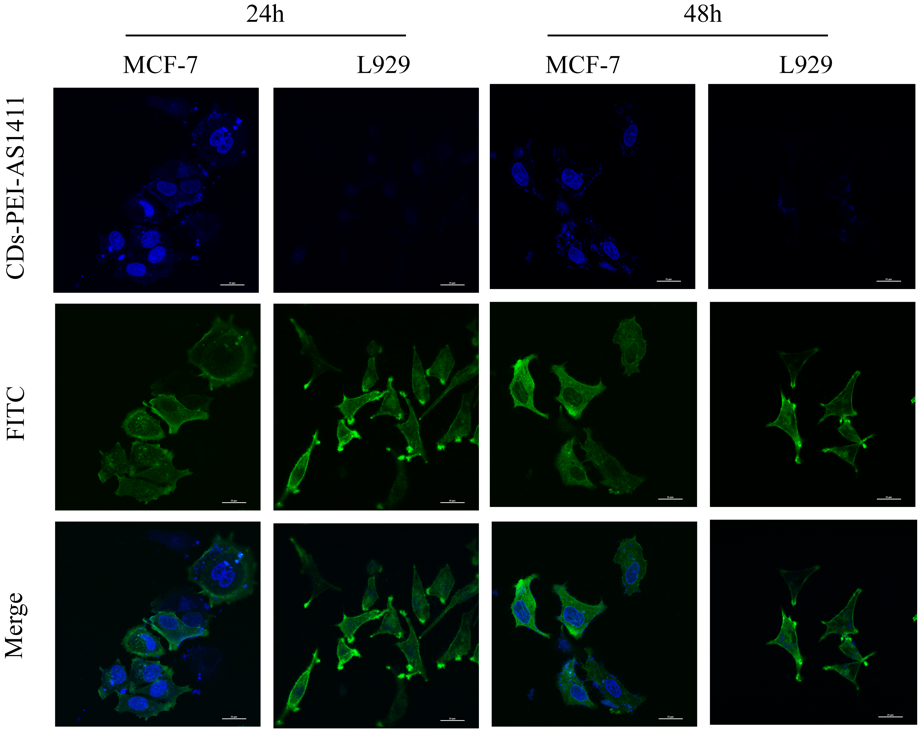


**Figure S2.** Confocal microscopy images of MCF-7 cells and L929 cells. The cells were incubated with the CDs-PEI-AS1411 for 24 h and 48 h.


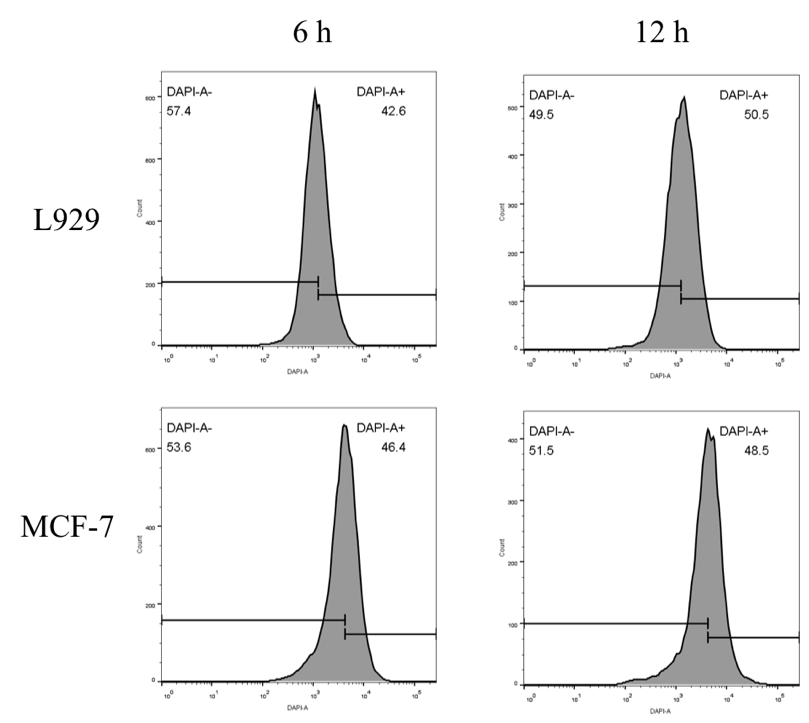


**Figure S3.** Flow cytometry profiles of L929 cells and MCF-7 cells treated with the CDs-PEI for 6 h and 12 h, respectively.
